# The Link Between Matrix Metalloproteinases and Alzheimer’s Disease Pathophysiology

**DOI:** 10.1007/s12035-024-04315-0

**Published:** 2024-06-27

**Authors:** Dominika Radosinska, Jana Radosinska

**Affiliations:** 1 Institute of Medical Biology, Genetics and Clinical Genetics, Faculty of Medicine, Comenius University in Bratislava, Bratislava, Slovak Republic; 2 Institute of Physiology, Faculty of Medicine, Comenius University in Bratislava, Sasinkova 2, 81372 Bratislava, Slovak Republic

**Keywords:** Alzheimer’s disease, Matrix metalloproteinases, Amyloid, Biomarker, Cell cultures, Human samples

## Abstract

Alzheimer’s disease (AD) is a major contributor to dementia and the most common neurodegenerative disorder. In AD pathophysiology, matrix metalloproteinases (MMPs)—proteolytic enzymes, best known to be responsible for remodeling and degradation of the extracellular matrix—were suggested to play an important role. Due to the diverse nature of the published data and frequent inconsistent results presented in available papers, it was considered essential to analyze all aspects of MMP literature with respect to AD pathophysiology and attempt to outline a unifying concept for understanding their role in AD. Thus, the main contribution of this review article is to summarize the most recent research on the participation of MMP in AD pathophysiology obtained using the cell cultures to understand the molecular principles of their action. Furthermore, an updated comprehensive view regarding this topic based exclusively on papers from human studies is provided as well. It can be concluded that determining the exact role of any particular MMPs in the AD pathophysiology holds promise for establishing their role as potential biomarkers reflecting the severity or progression of this disease or for developing new therapeutic agents targeting the processes that lead to AD.

## Introduction

### Neuropathology of Alzheimer’s Disease

**Fig. 1:**
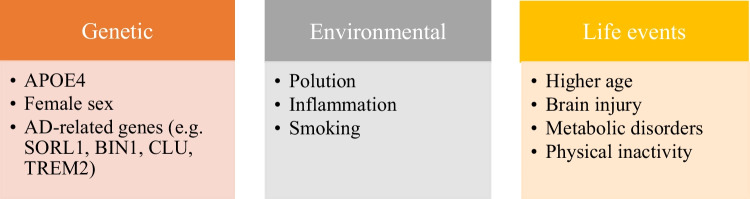
The main risk factors for the development of late-onset Alzheimer's disease (APOE4, apolipoprotein E4)

Alzheimer’s disease (AD) is a major contributor to dementia and the most common neurodegenerative disorder leading to brain atrophy as a consequence of neuronal degeneration and ultimately neuronal death. According to genetics, AD can be divided into two main types. Familiar or early-onset AD (FAD/ EOAD) is quite rare, with less than 5% of all AD cases. FAD usually occurs before the age of 65 and is caused by mutations in the following genes: amyloid precursor protein (APP), presenilin 1, or presenilin 2. These mutations are linked with an increased production of amyloid β (Aβ) peptides due to abnormal APP cleavage. However, the vast majority of AD cases are sporadic or late-onset AD (LOAD). LOAD has a multifactorial pathology that includes genetic risk factors, environmental factors, and life events [1] (Fig. 1). Being more specific, the main risk factors of LOAD development are higher age, female sex, traumatic brain injury, and inflammation, as well as the possession of one or two copies of the apolipoprotein E (APOE) ε4 allele. The APOE gene codes the ApoE glycoprotein, which is responsible for transporting lipoproteins, especially cholesterol and phospholipids to various cells via blood plasma. In the central nervous system (CNS), ApoE is important for the growth of neurons as well as their repair [2]. It is mostly secreted by astrocytes and microglia and occurs in the cerebrospinal fluid [3–5]. Human APOE occurs in three genetic isoforms—APOE2, APOE3, and APOE4. Possession of the APOE ε4 allele is considered to be the strongest genetic risk factor that reduces the mean age of LOAD onset compared with the ε3 and/or ε2 allele [6].

**Fig. 2:**
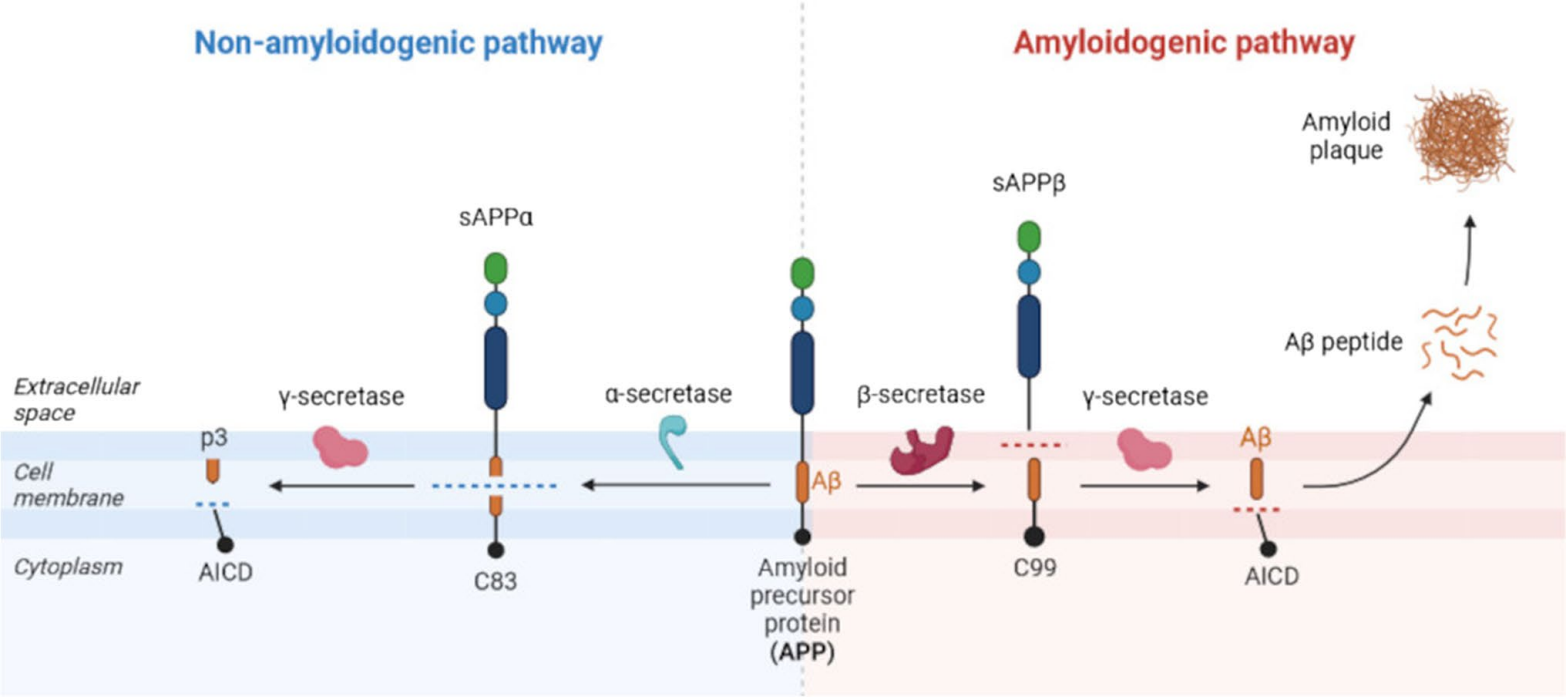
Processing of the amyloid precursor protein. Abbreviations: APP, amyloid precursor protein; sAPPβ, soluble N-terminal fragment APP β; C83/C99, C-terminal fragments of specified length; AICD, APP intracellular domain; Aβ, amyloid beta; sAPPα, soluble N-terminal fragment APP α; p3, peptide fragment. Adapted from “Cleavage of Amyloid Precursor Protein (APP),” by BioRender.com (2023). Retrieved from https://app.biorender.com/biorender-templates

The histologic hallmarks of AD include the occurrence of Aβ peptides and neurofibrillary tangles (NFTs). Extracellularly occurring Aβ peptides are cleavage products derived from APP. Their deposits in the brain parenchyma are also called amyloid or senile plaques. Intensively studied APP is a transmembrane glycoprotein of 110–130 kDa encoded by a single gene, located on chromosome 21, that generates various protein isoforms with different length of amino acid chains. The most known APP isoforms are APP695 that is predominantly expressed in neurons, as well as APP751 and APP770 expressed in glial cells [7]. The APP function has been assumed in neurite outgrowth and synaptogenesis, neuronal protein trafficking along the axon, transmembrane signal, and calcium metabolism [8]. APP processing is generally divided into two pathways, non-amyloidogenic and amyloidogenic (Fig. 2).

The non-amyloidogenic (or secretory) pathway starts with α-secretase, which releases the ectodomain, soluble N-terminal fragment APP α, and a C-terminal fragment of APP that is of 83 amino acid length (C83). Further cleavage of C83 is mediated by γ-secretase, releasing a small p3 fragment into the extracellular space and the APP intracellular domain into the cytoplasm. Conversely, the amyloidogenic APP processing is mediated by β-secretase, resulting in a smaller ectodomain—soluble N-terminal fragment APP β, and a longer C-terminal fragment (C99), which is subsequently cleaved by γ-secretase, generating the Aβ peptide and APP intracellular domain [9]. To date, the most extensively studied APP metabolites are the Aβ peptides, specifically Aβ40 and Aβ42 species in the brain. Their well-established neurotoxic effects due to overproduction leading to their aggregation and accumulation into amyloid plaques are associated with the development of AD. Initially, misfolded amyloid monomers are able to aggregate and generate unstable but still soluble oligomers. Further, these oligomers are assembled to form short, flexible protofibrils that finally mature into insoluble fibrils more resistant to proteolytic cleavage [10].

NFTs that occur inside the neurons represent accumulations of hyperphosphorylated tau protein. Tau protein is encoded by the microtubule-associated protein tau gene in human chromosome 17 and is found in neurons, specifically within an axonal compartment. It is also expressed in glial cells, mainly in pathological conditions—i.e., tauopathies [11]. Tau protein includes an N-terminal projection domain and a C-terminal domain, which contains the microtubule-binding region. The projection domain is responsible for interaction with other cytoskeletal elements and neuronal plasma membrane. The microtubule-binding domain is involved in the tubulin assembly, axonal transport, and the modulation of tau protein phosphorylation [12]. In pathological conditions—e.g., in AD, tau protein is abnormally hyperphosphorylated [13], and consequently can easily aggregate to form NFTs. Following these events, tau protein is unable to bind to microtubules, maintaining the cytoskeletal stability, that leads to impairment of axonal transport and synaptic metabolism [14]. Tau protein can also interact with other proteins during its post-translational modifications as well as proteins that affect its phosphorylation. One of these proteins is Aβ peptide. The research group of Liang et al. [15] suggested various interactions between tau protein and Aβ peptides. Aβ peptides promote the tau protein hyperphosphorylation by the activation of multiple kinases, as well as tau oligomerization and aggregation leading to NFTs formation. And conversely, tau protein has been shown to be capable of inducing Aβ toxicity [16].

### MMP—Classification, Structure, and Functions in CNS

Matrix metalloproteinases (MMPs) are a large family of structurally related zinc-dependent proteases. They belong to a larger superfamily of metzincins, which include also adamlysins and astracins. According to their structure, localization, and substrate specificity, MMPs have been divided into six groups: collagenases, gelatinases, matrilysins, stromelysins, membrane-type MMPs (MT-MMP), and the other MMPs. MMPs consist of the following domains: signal peptide, propeptide, catalytic, hinge region, and hemopexin-like domain. MMPs are produced like zymogens (pro-MMPs), which are inactive proenzymes that are converted into catalytically active proteases by the mechanism known as cysteine switch. After activation, MMPs are involved in various physiological and pathophysiological processes, such as extracellular matrix (ECM) remodeling, development, morphogenesis, inflammation (activation or inactivation of multiple inflammatory mediators), and signaling pathways [17–19].

An activity of MMPs must be strictly regulated at multiple levels, such as gene expression, activation of the pro-MMPs by a proteolytic cleavage, production of the endogenous inhibitors—i.e., tissue inhibitors of metalloproteinases (TIMPs) as well as by other non-specific inhibitors—e.g., α2-macroglobulin or plasma inhibitor [20]. In addition, MMPs can be up-regulated and simultaneously activated by various stimuli including reactive oxidative species, cytokines, and growth factors. In inflammatory conditions, MMP deregulation can be involved in blood–brain barrier (BBB) disruption, neurotoxicity, breakdown of tight junctions, and abnormal cleavage of ECM components (e.g., collagen, laminin). This has been observed in a variety of pathological conditions, including neurodegenerative processes, cancers, metastases, infections, immune diseases, atherosclerosis, and many others [19, 21–23].

**Fig. 3:**
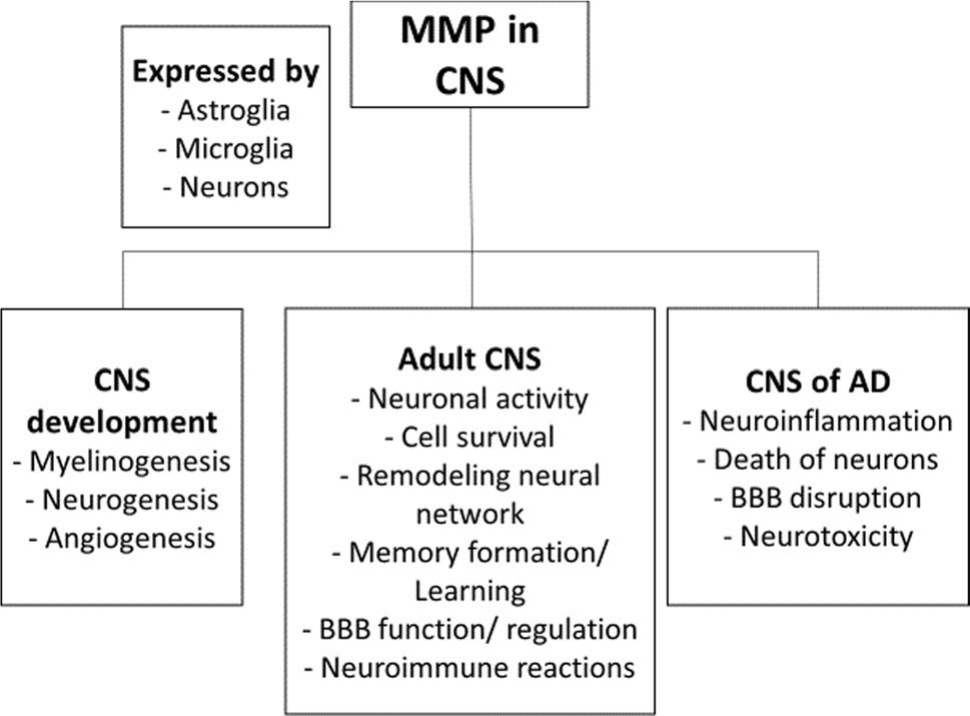
Multiple functions of matrix-metalloproteinases in the central nervous system. Abbreviations: MMPs, matrix-metalloproteinases; CNS, central nervous system; BBB, blood–brain barrier; AD, Alzheimer’s disease

Regarding the role in the regulation of CNS function (Fig. 3), MMPs have so far been shown to be involved in the regulation of remodeling neural network, neuron-glial cell intercommunication, neuroinflammation, neuroimmune reactions, BBB function, neuronal activity, survival, and death [24]. In the CNS, various MMPs are expressed by astroglia and microglia, as well as by neurons [21]. MMPs are also important in CNS development—particularly in myelinogenesis, neurogenesis, axonal guidance, and growth, as well as angiogenesis [25]. However, their role was also recognized in the healthy adult CNS. Under normal conditions, ECM regulation by MMPs participates in cell migration and survival, synaptic plasticity, learning, and memory, as well as significantly contributes to tissue repair [25]. When an imbalance between the MMP formation and their inactivation occurs, it can lead to neurodegeneration, promoting diseases such as AD [23]. The research group of Crocker et al. was focused on MMPs expressed by astrocyte and microglia cultures. They demonstrated that microglia, but not astrocytes, are the major source of MMP-9 after the exposure to pro-inflammatory molecules, such as lipopolysaccharide or tumor necrosis factor-α. In contrast, the astrocyte culture was shown to constitutively express MMP-2, -11, and -14. In addition, astrocytes showed a small induction of MMP-3 following the stimulation by interleukin-1β. These observations could be relevant in AD research, as neuroinflammation to a high extent participates in the progression of this disease [26].

In further sections, the research done using cell cultures and data regarding the MMP activities and levels in plasma, cerebrospinal fluid, and postmortem tissue of AD individuals is summarized to highlight the participation of MMPs, as well as their inhibitors, in AD pathophysiology. While numerous review papers have explored the connection between MMPs and the pathophysiology of various neurodegenerative diseases, this review specifically concentrates on AD, rather than covering a broader range of neurological conditions. The primary focus of this review is to summarize the latest research on MMP involvement in AD pathophysiology, particularly highlighting findings obtained from cell culture studies to elucidate the molecular mechanisms of their action. Additionally, this review offers an updated and comprehensive perspective on this topic, drawing exclusively from human studies, with a particular emphasis on distinguishing between MMP activities and concentrations.

## Cell Cultures, MMPs, and AD

To elucidate the link between MMPs and AD pathophysiology, multiple research teams examined various potential mechanisms using cell cultures. Such an approach still represents one way to improve our knowledge about the role of MMPs in different neurodegenerative disorders, including AD.

The majority of studies showed an upregulation of soluble MMPs (i.e., those secreted by cells into extracellular space) in the presence of Aβ peptides. Aβ peptides can trigger an increase in MMP-9 (but not MMP-2) activity in rat astrocytes [27] as well as in monocytic cell cultures after the stimulation by IFN-γ [28]. Interestingly, such stimulation of the same cell culture was achieved after the incubation of cells with plasma from AD patients, but not from patients with mild cognitive impairment (MCI) [29]. Using HEK-293 cells transfected with APP695 and MMP-9, degradation promoting the production of soluble N-terminal fragment APP α (generated by α-secretase, but not β-secretase) was shown, simultaneously with reduced production of Aβ peptides [30]. Taking into consideration these results, MMP-9 may inhibit the formation of amyloid plaques and thus could offer neuroprotection. This idea is supported by an observation of MMP-9 ability to digest Aβ fibrils in vitro and compact plaques in vivo [31]. Additionally, various cell-conditioned media were able to degrade Aβ40 and Aβ42 peptides, while this ability was attenuated after the inhibition of MMP-2 and MMP-9 [32, 33]. The difference in the proteolytic activity between recombinant MMP-2 and MMP-9 was also observed during the cleavage of Aβ peptides in human neuroblastoma cells and human cerebral microvascular endothelial cells, where MMP-9 was significantly less effective in the degradation of Aβ40 as compared with MMP-2, while Aβ42 was cleaved only by MMP-2 [34]. The study of Deb et al. focused on the effect of monomeric (i.e., freshly prepared) and aggregated fibrillar (i.e., aged) Aβ40 and Aβ42 peptides on MMP induction and activity in rat astrocytes. They demonstrated that freshly prepared and aged Aβ peptides differently stimulated the activity of gelatinases. Thus, Aβ cleavage property of different MMPs may not be equally effective in any Aβ peptide condition. In addition, MMP-2 activity was suppressed at the highest level of aged Aβ42 peptide [35]. This observation seems to be consistent with another finding reporting a decrease in MMP-2 activity as well as its expression after incubation with fibrillar Aβ42 peptides in primary astrocyte culture [36]. There is also a study focused on the effect of Aβ(25–35) fragment on neuronal and astrocyte, as well as mixed cortical cultures. Incubation of cells with this fragment induced different effects in different cultures—i.e., an activation of MMP-9 and MMP-2 in astrocytes, while increased production of TIMP-1 by neurons. The changes in release of these molecules were accompanied by modifications in their gene expression. In addition, Aβ(25–35) fragment induced toxicity only in cultured neurons, whereas it stimulated proliferation in astrocytes that was shown at least partially caused by enhanced TIMP-1 release by neurons [37]. An overview of observations regarding the link between MMPs and AD pathophysiology using different cell cultures is presented in Table 1.

**Table 1:** Changes of selective MMPs/TIMPs in different cell cultures under AD conditions

Cell culture	Findings related to AD	Reference
HEK-293—expressing APP695 and MMP-9	↑sAPPα and ↓Aβ40	[30]
Microglia	+ Aβ42 and NO donor →↓TIMP-1 and ↑MMP-9 →↑proteolysis of Aβ peptides	[38]
Astrocytes	+ Aβ40 →↑MMP-9 (but not MMP-2)	[27]
	+ Aβ40/42 →↑MMP-9	[35]
	+ Aβ42 →↓MMP-2	[36]
	GPR120 inhibition →↑MMP-2/-14, ↓TIMP-3/-4 and ↑Aβ proteolysis	[39]
	+ Aβ(25–35) →↑MMP-9/-2	[37]
Human brain microvascular endothelial cells	+ Aβ42 →↑MMP-9	[40]
	+ Aβ42 → cleavage by MMP-2	[34]
	+ Aβ40 → cleavage by MMP-9/-2	[34]
Monocytes	stimulation by IFN-γ + Aβ40 →↑MMP-9	[28]
Human neuroblastoma cells	+ Aβ42 → cleavage by MMP-2	[34]
	+ Aβ40 → cleavage by MMP-9/-2	[34]
	+ Aβ40 →↑MMP-9	[30]
Neurons	+ Aβ(25–35) →↑TIMP-1 (protein and gene)	[37]
COS-1—expressing MMP-14	↑proteolysis of Aβ peptides	[41]

Abbreviations: ↑ –increased, ↓ –decreased, + –addition or incubation with the particular type of amyloid peptide, sAPPα – soluble N-terminal fragment of amyloid precursor protein α, GPR120 – G-protein coupled receptor 120, IFN-γ – interferon γ, NO – nitric oxide, AD – Alzheimer’s disease, Aβ – amyloid β, MMP – matrix metalloproteinases, TIMP – tissue inhibitors of metalloproteinases

By the use of cell cultures, the effect of different ApoE isoforms on MMP activity was demonstrated as well. More specifically, astrocytes co-incubated with fresh Aβ40 and ApoE4 showed lower MMP-9 activity in comparison with ApoE2 [27]. Another work also described a stimulation of MMP-9 release in the presence of Aβ42 peptides using primary human brain microvascular endothelial cells. Moreover, the effect of MMP-9 on the elimination of Aβ peptides across the BBB dependent on lipoprotein receptors was studied. In vitro experiments demonstrated that the overall process is influenced by ApoE in an isoform-specific manner. ApoE3 was linked with preservation of the lipoprotein receptor transport, and consequent Aβ peptide elimination, while ApoE4 may via lipoprotein receptor shedding reduce the clearance of Aβ peptides and in this way promote AD burden [40]. The same research group studied the effects of individual ApoE isoforms on MMP-9 secretion by the same cell line. The activity of MMP-9 was higher in co-incubation with ApoE4 and ApoE3, while the ApoE2 was the most effective in inhibiting MMP-9 cell secretion [42]. Not only MMPs but also their inhibitors could be modified differently depending on ApoE isoform as another study detected increased levels of TIMP-1 in astrocyte cultures from mice with targeted gene replacement of murine APOE with human APOE4, but not APOE3 [43].

In search of the mechanisms responsible for MMP activation, it has been shown that MMP-9/TIMP-1 balance is regulated by nitric oxide (NO). Microglia cells treated by NO donor exhibited a decreased level of TIMP-1. Consequently, the increased ratio of MMP-9/TIMP-1 and, particularly, an increase in MMP-9 activity was responsible for enhanced proteolysis of Aβ42 peptides in the presence of NO [38]. Another study focused on the effect of G protein–coupled receptor (GPR120) signaling on Aβ degradation in primary astrocytes. GPR120, being receptor for omega-3 polyunsaturated fatty acids, was shown to exert anti-inflammatory responses. An inhibition of GPR120 resulted in an enhancement of MMP-dependent Aβ-degrading activity. Although an increase in the gene expression of MMP-2 and -14, concomitantly with decreased expression of TIMP-3 and -4 was shown, MMP-14 was identified as crucial for Aβ cleavage in these experimental settings [39]. While MMP-2 and -9 are soluble, MMP-14 (also referred to as MT1-MMP) is anchored in the cell membrane.

In databases, there are more studies focused on membrane-bound MMPs (Table 2). For example, an enhanced degradation of exogenous Aβ40 and Aβ42 was detected by the use of COS-1 cells expressing human MT1-MMP (= MMP-14) [41]. Another research investigated the cleavage of APP by multiple MT-MMPs, such as MT1-MMP, MT3-MMP, and MT5-MMP in HEK-293 T and COS-1 cells. The shedding of APP fragments was the most effective by MT3-MMP, then MT1-MMP and finally MT5-MMP. It was confirmed that MT3-MMP might cleave APP within the Aβ peptide sequence in the same way as α-secretase possibly preventing accumulation of amyloid [44]. MT1-MMP has been shown as a regulator of APP proteolysis induced by oxidative stress in human neuronal cells. Oxidative stress induced an accumulation of the 85 kDa N-terminal APP fragment (APP85) and simultaneously an activation of MT1-MMP, which was found preferentially in the lysosome fraction. Thus, it was suggested that MT1-MMP could be responsible for APP processing, as its inhibition prevented the accumulation of APP85 under oxidative stress conditions [45].

**Table 2:** The suggested role of MT-MMPs in AD pathophysiology by the use of cell cultures

	Findings related to AD	Reference
MT1-MMP	Degradation of Aβ40/42	[41]
	A regulator of APP proteolysis under oxidative stress conditions	[45]
	In FAD/EOAD—its overexpression →↑C99 → amyloid formation	[46]
MT3-MMP	APP cleavage, like α-secretase	[44]
MT5-MMP	In FAD/EOAD - Its overexpression →↑C99 → amyloid formation	[47, 48]
	- Colocalized to early endosomes	[49]
	- Specific domains have differential impact on APP metabolism	[50]
	- Catalytically active or inactive fragments →↑Aβ40	[50]
	- Can be modified by AD transgenes →↑protein levels	[51]
	- With IL-1β, IL-6, MCP-1 → promote neuroinflammation	[51]
	- Colocalized to early endosomes	[51]

Abbreviations: FAD/EOAD – familiar Alzheimer’s disease/early-onset Alzheimer’s disease, ↑ –increased, AD – Alzheimer’s disease, Aβ – amyloid β, MT-MMP – membrane-type matrix metalloproteinases, C99 – C-terminal fragment of amyloid precursor protein, IL – interleukin, MCP-1 – monocyte chemoattractant protein-1

There are additional studies documenting the role of MT-MMP in cell cultures, which relate to early onset of AD. In HEK cells expressing the APP-Swedish mutation (= HEKswe cells), MT1-MMP overexpression increased the production of APP C-terminal fragments—C99, indicating APP cleavage that favors amyloid formation [46]. Thus, the potential of MT1-MMP as a pharmacological target of AD was suggested. Moreover, they examined another membrane-bound MMP—i.e., MT5-MMP, overexpressed in HEKswe cells. Like MT1-MMP, MT5-MMP was also shown to be colocalized with APP, responsible for increase in C99 and Aβ production [47, 48]. Regarding the intracellular trafficking of APP (promoting the amyloid formation), MT5-MMP was found localized to early endosomes [49]. The more recent study of the same research group focused on different MT5-MMP domains that specifically affect APP metabolism and Aβ production in HEKswe cells. Both catalytically active and inactive fragments MT5-MMP increased the extracellular Aβ40 levels, suggesting that the C-terminal region of MT5-MMP alone could promote amyloidogenesis [50]. In mixed neuron/astrocyte cultures obtained from mouse model of familiar AD (expressing five mutations in human AD transgenes—i.e., 5xFAD mice), increased protein levels of MT5-MMP (but not its mRNA) were observed, documenting modifying effects of AD transgenes on this membrane-bound MMP. In addition, an interaction between MT5-MMP and pro-inflammatory molecules (e.g., IL-1β, IL-6, MCP-1) was shown to be important in early AD pathogenesis, as MT5-MMP-deficient cells showed a decrease in neuroinflammation, APP/Aβ metabolism, and neuronal excitability [51].

Although cell culture studies do not always strictly reflect in vivo conditions, based on published results, it may be hypothesized that soluble MMPs—e.g., gelatinases (MMP-2 and MMP-9), by themselves do not contribute to AD pathophysiology. It was suggested that gelatinases might be beneficial in terms of cleavage of Aβ products by both MMP-2 and MMP-9 rather than their active contribution to AD pathogenesis. However, in general they are increased in AD conditions, and thus can serve as biomarkers mirroring the AD severity. It is also applicable to MT-MMPs, except in experiments using cell cultures linking to the familiar early onset of AD (i.e., FAD). In this case, MT-MMPs were shown to be involved in the formation of toxic APP metabolites, thus emerging as a potential therapeutic target in AD.

## Brain Tissue, MMPs, and AD

In addition to research by using cell cultures that could contribute to knowledge regarding the mechanisms of MMP regulation in various conditions related to AD pathophysiology, we can also get data about the possible role of MMPs in AD disease studying the brain tissue obtained postmortem from AD patients. The summary of changes in MMP activity or MMP concentration in individual CNS regions is available in Table 3.

**Table 3:** Alteration of specific MMPs in different brain regions of AD patients

	Change in AD, brain locality	References
MMP-1	↑ In frontal, temporal, parietal, occipital cortex	[52]
MMP-9	↑ In hippocampus ↑ In cerebral vessels ↑ In frontal, parietal cortex	[53, 54, 56, 59] [42] [57]
MMP-3	↑ In amyloid plaques in hippocampus, parietal cortex ↑ In prefrontal cortex	[55] [62]
MMP-2	↑ In hippocampus (in late stages of AD)	[59]
	↑ In entorhinal cortex (in early stages of AD)	[61]

Abbreviations: AD – Alzheimer’s disease, MMP – matrix metalloproteinase

The involvement of selected MMPs in AD pathophysiology is also supported by the observation of higher MMP-1 protein concentrations in several cortical areas (i.e., in the frontal, temporal, parietal, and occipital cortex) in AD patients compared with age-matched cognitively normal elderly individuals [52]. In the hippocampus of AD patients, an increase in the gelatinolytic activity of high-molecular-weight proteinases corresponding to pro-MMP-9 was detected as well [53]. Pro-MMP-9 was shown to be expressed by hippocampal pyramidal neurons. After its activation, this proenzyme form was able to cleave Aβ40 peptides at several sites of the amino acid sequence [54]. Another MMP—MMP-3—was shown to be predominantly expressed in the white matter of the parietal lobe and hippocampus with no differences between controls and AD patients. However, MMP-3 was absent in the corresponding cortex of control brains while visualized in amyloid plaques in the cortex of AD patients. Comparison of MMP-3-positive amyloid plaques showed a higher density in the parietal cortex than in the hippocampus [55]. MMP-9 (gelatinase B) was found in the cytoplasm of neurons, NFTs, amyloid plaques, and vascular walls in samples taken from the hippocampus as well as the parietal lobe of AD patients [56]. Another research group focused on the activities of both gelatinases (MMP-2 and MMP-9) in the frontal and parietal cortex in individuals with AD, MCI, and healthy controls. Pro-MMP-9 and MMP-9 activities were increased in frontal and parietal cortex samples from MCI and AD groups compared with controls; however, MMP-2 activities were not different among all three groups. Moreover, both pro-MMP-9 and MMP-9 activities negatively correlated with scores in screening tests of cognitive functions [57]. Noteworthy, MMP-9 activity has been shown higher in comparison with MMP-2 activity in the brain tissue of AD patients as well as controls [58]. This fact is also supported by another study that examined the relationship between MMP-2/-9 and tau protein levels in individuals with early, moderate, and late AD. Not only activities, but also protein levels of MMP-9 were higher compared with MMP-2 in hippocampal samples. In addition, while the MMP-9 protein level increased in the case of moderate and late AD, the MMP-2 protein level was higher only in late AD when compared with controls [59]. However, a study focused on the entorhinal cortex (the brain locality, which is crucial for memory formation and recall, and critically affected in AD conditions [60]) reported increased MMP-2 activities as well as concentrations in the early stages of AD [61] suggesting differential modulation of MMPs in different brain regions in AD pathology. In the prefrontal cortex, an expression and level of MMP-3 were increased in individuals with MCI and AD. In addition, MMP-3 protein concentration negatively correlated with the score in Mini-Mental State Examination (MMSE) and positively with an occurrence of amyloid deposits and NFTs [62]. It was also suggested that an increased expression of MMP-9 in moderate and late AD cases participated in the post-translational modifications of tau protein during the formation of the NFTs. Specifically, the co-localization between MMP-9 and tau protein was detected in the intracellular NFTs. MMP-9 might have had a significant role at the beginning of neurodegenerative pathology, i.e., before tau protein aggregated [59]. However, the cleavage of tau protein by MMP-9 was limited and resulted in increased tau aggregation during in vitro conditions, whereas a reduction of tau aggregation was observed in the presence of high concentrations of MMP-3 [63]. Besides MMPs, TIMPs were also found to be localized near the amyloid plaques and NFTs in AD brain samples [64]. Compared with controls, total and active MMP-9 levels in AD patients were significantly higher also in the cerebrovasculature of human brain tissue. In an analysis of MMP-9 activity associated with the APOE genotype, the APOE4 genotype (APOE3/4 and APOE4/4) in AD individuals was related to markedly increased active MMP-9 levels compared with control subjects [42]. In contrast, another study showed no differences in the distribution, concentrations, and activities of MMP-2, -3, and -9 and their relation to Aβ load in the frontal cortex of AD and control groups [65].

## Body Fluids, MMPs, and AD

Determining the activities/concentrations of MMPs as well as their inhibitors in various body fluids of AD patients is likely to be of most interest to those active in AD research in clinical settings.

### Blood Plasma

An overview of data regarding the activity and concentrations of MMPs and TIMPs measured in plasma/serum is available in Table 4.

**Table 4:** Activity and concentration of MMPs and TIMPs measured in plasma/serum of patients with Alzheimer’s disease

	Activity	References	Concentration	References
MMP-1			-,-,↓,-,↓	[66–70]
MMP-2	-,-,-,↓,-	[70–74]	-,-,↑,-,-,-,↓	[66, 68, 70, 73–76]
MMP-3	↑,↑	[72, 77]	-,↑	[68, 69]
MMP-7			-	[66]
MMP-8			↓	[78]
MMP-9	↑,-,↓,-,↑	[70–74]	↑,-,-,↑,↑,-,-,-,-,-	[66, 68–70, 74–76, 79–81]
MMP-10	-	[72]	↓,-,-	[66, 68, 75]
TIMP-1			-,-,-,-,↓,-	[66, 70, 74–76, 79]
TIMP-2			-,-,-,-	[70, 74–76]
TIMP-3			↓	[82]
TIMP-4			↑	[78]

Abbreviations: MMP – matrix-metalloproteinase, TIMP – tissue inhibitors of MMPs, ↑ – higher value in AD patients in comparison with controls, ↓ – lower value in AD patients in comparison with controls, “-“ – no difference between AD patients and controls

In blood plasma, MMP-1 concentration was found either unchanged [66, 70] or decreased [67, 68] in AD patients when compared with controls. Similar findings were reported regarding the MMP-2 activity—i.e., it was either unchanged [70–72, 74] or decreased [73] in plasma of AD patients in comparison with controls. The available data on MMP-2 concentration are even more inconsistent. Although there was no statistical difference between AD patients and controls in most studies [66, 70, 73–75], there are also studies reporting its decreased [76] as well as increased [68] value. The activity of MMP-3 was detected consistently higher in AD patients [72, 77], while its concentrations were not modified [68]. MMP-7, which has been shown to be able to cleave Aβ peptides during in vitro conditions [83], was not significantly different between AD and control individuals [66]. Focusing on MMP-9 activities, inconsistent data were reported, probably due to the presence of various comorbidities affecting this MMP signaling [19, 84, 85]. MMP-9 level, which was detected higher in neuronally derived extracellular vesicles in plasma of AD patients [86], was found to be higher in plasma samples of these patients only in some of the available studies [70, 74, 81]. Plasma MMP-9 concentration did not differ between AD patients and controls in most reports [66, 68, 75, 76, 79, 80]. The plasma concentration of TIMP-1 and TIMP-2 was generally not different in AD patients compared with controls as well [66, 70, 74, 75, 79]. Just one study reported lower TIMP-1 with unchanged TIMP-2 concentration in AD patients [76].

Remarkable data were noted when results on MMPs and TIMPs were examined in various relationships. Despite the TIMP-1 and TIMP-2 levels did not differ between patients and controls, their positive correlation with MMP-9 concentrations was revealed in the AD condition [74]. The level of MMP-9, which was found to be elevated in AD patients compared with controls, was also significantly elevated compared with MCI individuals [70]. The relation between the oxidative stress markers and MMPs was also observed. MMP-2 and MMP-9 levels were not significantly different between AD or MCI patients and controls, but they negatively correlated with oxidative stress marker (malondialdehyde) in AD and MCI samples [71]. MMP-2 activity, which was found to be lower in AD samples compared with control subjects as well as MCI group, was positively correlated with the clinical data, such as the age and MMSE score [73]. MMP-3 activity, which was significantly higher in AD patients compared with the controls, was also negatively correlated with the MMSE test in AD patients [77]. In the study where MMP-2 and TIMP-1 levels were lower in plasma samples of AD patients compared with controls, the ratio of MMP-2/TIMP-2 was also significantly lower in AD patients than in controls. In addition, the levels of MMP-2 and MMP-9 positively correlated with TIMP-1 and -2 levels, and MMP-2 levels positively correlated with MMP-9 in the plasma of AD patients [76]. Another study was focused on longitudinal changes in MMP levels in plasma of MCI-AD patients. In this study, the ratio of the hippocampal volume to intracranial volume (H/ICV ratio) was monitored to estimate the degree of neurodegeneration. A correlation between baseline MMP-1, -9, and TIMP-1 levels and longitudinal changes in the H/ICV ratio at each annual visit was observed. Additionally, baseline MMP-1, -9, and -10 levels correlated with longitudinal changes in the MMSE score, as well as MMP-9 correlated with longitudinal changes in the AD assessment scale (ADAS)-11 score. The H/ICV ratio and tests of cognitive function (MMSE and ADAS-11) declined faster in those MCI-AD patients who had high levels of MMP-9 compared with patients with middle and low MMP-9 levels [66]. Circulating MMP-9 levels, along with other BBB-related biomarkers, have been investigated in relation to cognitive functions in AD patients. Although there was no significant difference between MMP-9 levels in the AD and control individuals, MMP-9 concentration was suggested as a promising biomarker of cognitive deficit [80]. It has been also suggested that sex might modulate MMP levels in AD patients as the incidence of AD is higher in women. Indeed, MMP-9 levels negatively correlated with the deterioration of cognitive functions estimated by the use of MMSE score only in female AD patients, although they were not different between men and women with AD. Moreover, higher MMP-9 concentration predicted a faster worsening of cognitive functions only in women [87]. However, estrogens might activate neuronal MMP-2 and MMP-9, which can degrade amyloid peptides in cell culture [88], so their increased activation may represent a compensatory mechanism rather than evidence of their undesirable contribution to AD pathophysiology. The research on angiogenesis in AD has identified an increase in TIMP-4 plasma levels in mild to severe forms of the disease. Moreover, TIMP-4 concentration was significantly associated with the higher risk of AD, and its levels were negatively associated with the score in MMSE [78].

### Cerebrospinal Fluid

In addition to plasma samples, multiple MMPs and TIMPs were analyzed in CSF—the data overview is presented in Table 5. There appears to be a general agreement regarding the MMP-10 concentration that was found elevated in the CSF of AD patients in comparison with controls [68, 75, 89–93]. The higher level of MMP-10 in CSF of MCI-AD patients was identified as biomarker discriminating this group from patients with other neurological disorders (headache, psychiatric disorders, and mononeuropathy). Moreover, MMP-10 negatively correlated with CSF Aβ42 levels within MCI-AD group [91]. MMP-10 level in CSF was proposed to be a new promising biomarker for diagnosis of early-stage AD [94]. Consistently, CSF MMP-10 concentration was suggested as a biomarker reflecting the progression of MCI to AD dementia [92]. The proteomic study of CSF revealed significant biochemical similarities between sporadic and familiar AD. In both types, several new proteins, including MMP-10, were identified, indicating their potential as novel biomarkers [95]. In addition to MMP-10, a broader series of AD biomarkers was analyzed in MCI-AD individuals. There is a study focused on the association of MMPs and TIMPs with tau-/amyloid pathology. Data analyses showed that while tau pathology is strongly associated with MMPs (MMP-2,-3,-10) and TIMPs (TIMP-1,-2,-3,-4), amyloid pathology is only associated with MMP-3 and TIMP-4 levels. The authors proposed that alterations in the concentrations of MMPs and TIMPs are more closely linked to tau pathology in comparison with amyloid pathology. Furthermore, sex differences were observed, with amyloid pathology being associated with MMP-3 in men and with TIMP-4 in women [89]. However, when the levels of MMP-3 in the CSF decrease and its normal functions thus deteriorate, it could lead to more pronounced age-related brain atrophy. This phenomenon has been observed in individuals without cognitive impairments and appears to be independent of CSF Aβ42 levels [96]. Another study analyzed MMP-1, -3, and -9 and TIMP-1 levels in patients with AD and controls. An increased MMP-9/TIMP-1 ratio in AD patients compared with controls indirectly suggested promotion of MMP-9 activity in the AD brain [97]. However, according to other studies that were focused on direct measurements of MMP activities, MMP-9 activity in the CSF of AD patients was either not detectable [72, 98] or not modified compared to controls [99]. On the other side, higher MMP-9/TIMP-1 ratio was associated with higher tau concentration in CSF of AD individuals [97]. MMP-3 levels as well as MMP-3/TIMP-1 ratios were increased in AD patients compared with controls (i.e., individuals with subjective cognitive impairment). Furthermore, MMP-3 levels positively correlated with total and phosphorylated tau (t-tau and p-tau) levels, and negatively correlated with MMSE score [98]. In the group of individuals with mild to moderate AD dementia, no differences were found in MMP-1, MMP-2, and MMP-9 protein levels, as a function of patient APOE genotype, but the ratio of active MMP-9 to its pro-form was significantly lower in APOE4/E4 CSF samples. In addition, APOE4/E4 patients also have higher TIMP-1 and TIMP-3 protein levels when compared with APOE3/E3 and APOE3/E4 AD individuals [43]. Furthermore, control individuals with a higher risk of AD (CSF Aβ42/p-tau ratio < 6.5 combined with CSF T-tau > 350 ng/l) had elevated levels of MMP-9. Interestingly, controls with the APOE ε4 allele had increased MMP-3 and -9 levels compared with non-carriers’ controls [97].

**Table 5:** Activity and concentrations of MMPs and TIMPs measured in cerebrospinal fluid of patients with Alzheimer’s disease

	Activity	References	Concentration	References
MMP-1			-, nd,↑	[68, 90, 97]
MMP-2	↓,-,-	[72, 98, 99]	-,-,-,-,-,-,-	[68, 75, 89, 90, 98, 101, 102]
MMP-3	↑ (p = 0.053)	[72]	-,-,-,↑,-,↑,↑	[68, 89, 90, 97, 98, 101, 103]
MMP-7			nd	[98]
MMP-9	nd, nd, -	[72, 98, 99]	-,-,-,-,-,↓	[68, 75, 90, 97, 98, 101]
MMP-10	nd	[72]	↑,↑,↑,↑,↑,↑,↑,↑,↑	[68, 75, 89–93, 102, 103]
TIMP-1	-	[99]	-,-,↓,-,-,-	[75, 89, 90, 97, 98, 101]
TIMP-2	-	[99]	-,↑,-,-	[75, 89, 90, 101]
TIMP-3			↓,-	[82, 89]
TIMP-4			-	[89]

Abbreviations: MMP – matrix-metalloproteinase, TIMP – tissue inhibitors of MMPs, ↑ – higher value in AD patients in comparison with controls, ↓ – lower value in AD patients in comparison with controls, “-“ – no difference between AD patients and controls, nd – not detectable

The research, which focused on eight MMPs (MMP-1, -2, -3, -7, -8, -9, -10, and -13), divided the study participants into seven clusters according to their age, as well as Aβ peptide, t-tau, and p-tau levels. Interestingly, MMP-2 and MMP-3 levels were lower in the cluster characterized by very low Aβ levels, suggesting a role for these particular MMPs in the Aβ clearance into the CSF, contributing to the amyloid accumulation in brain tissue [100].

### Blood Plasma and Cerebrospinal Fluid

There are also available studies reporting simultaneous determination of MMP activities/levels in plasma and CSF. Horstmann et al. found that MMP-2 activity, which was not different in plasma between AD patients and controls, was significantly lower in CSF of AD patients. On the other side, MMP-3 activity was significantly higher in plasma, showing the same tendency in CSF of AD patients compared with controls. Authors of this study were unable to detect MMP-9 and MMP-10 activity in CSF. However, MMP-9 activity was significantly decreased in the plasma of AD patients, while MMP-10 activity was not different between the two groups—patients and their controls [72]. Further research—studying various neuroinflammatory molecules that could promote pathophysiologic processes in AD patients—found that concentrations of tau protein, MMP-1, and -10 are higher in CSF samples from AD patients compared with controls. However, MMP-1 was lower, and MMP-2 was higher in plasma of AD patients. Moreover, MMP-10 measured in CSF positively correlated with age and negatively with memory score of AD patients. MMP-2 negatively correlated with the most cognitive features such as attention, executive, language, and overall condition in plasma of AD patients. MMP-3 positively correlated with age in plasma samples [68]. Another multiplex proteomic study found increased plasma MMP-9 levels in MCI patients compared with amyloid-negative cognitively normal elderly controls. However, MMP-10 was significantly higher in the CSF of AD and MCI amyloid-positive patients. Additionally, CSF levels of MMP-10 were negatively associated with the cortical thickness values [93].

## Sex Differences in AD with a Focus on MMP/TIMPs

Sex differences in AD encompass variations observed in the prevalence, risk factors, symptom manifestation, and disease progression between males and females with multiple underlying mechanisms proposed [104, 105]. Consistently, these distinctions extend to MMP/TIMP pathways. For instance, higher levels of total and free MMP-9 were shown to be associated with elevated CSF t-tau in women but lower in men, suggesting a potentially greater impact of MMP-9 on AD-related pathological and cognitive changes in women. [87]. Moreover, age consistently correlated with increased CSF levels of MMP-2, TIMP-1, and TIMP-2 in both sexes, whereas MMP-3, MMP-10, and TIMP-4 levels were influenced solely in women. In females, the APOE ε4 genotype showed no association with MMPs or TIMPs, while in males, carriers of the APOE ε4 allele had increased levels of MMP-10, TIMP-1, TIMP-2, and TIMP-3 compared with non-carriers [89]. Furthermore, in blood plasma, MMP-3 levels were higher in men than women, with a sex-specific difference noted in the association between plasma MMP-3 levels and cognitive decline—lower MMP-3 was associated with less cognitive decline in women, but not in men [69]. Understanding these sex differences in AD is crucial for developing more personalized approaches to AD prevention, diagnosis, and treatment. However, more research is needed to elucidate the underlying mechanisms driving these differences.

## Methodological Approaches in AD Research Focused on MMPs

**Fig. 4:**
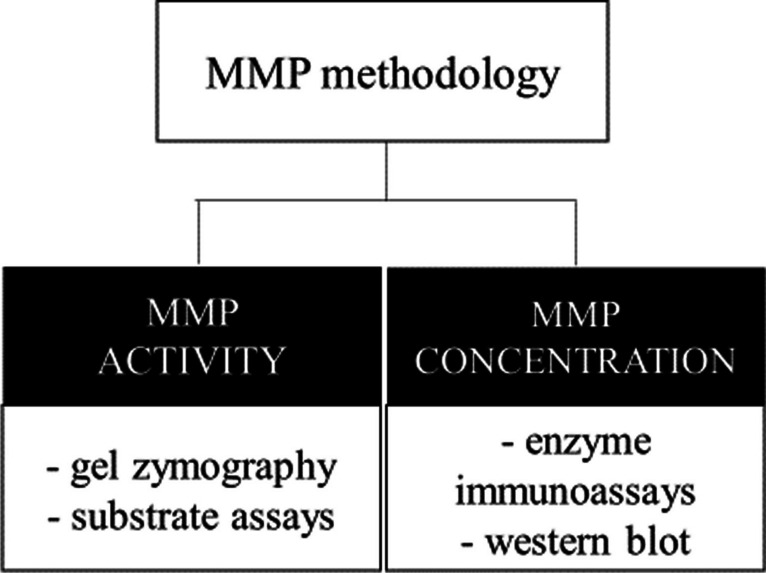
The most common methodological approaches in MMP (matrix-metalloproteinase) research

Generally, either MMP activities or MMP concentrations can be determined in various biological materials. In AD research, a vast majority of studies focused on MMP estimation in cell cultures (directly in cells or in their media), then in the brain tissue of experimental animals or humans, as well as in plasma or CSF samples. However, what should be taken into consideration is the difference between MMP activity and its concentration. While the concentration can remain stable, the MMP can be more or less active, resulting in a significant difference in terms of the final effect. On the other hand, the MMP concentration can vary, but resulting MMP activity can stay the same. Thus, it is crucial to apply different methodological approaches to get complete information regarding the possible role of a particular MMP (Fig. 4) in the pathophysiology of any disorder. For MMP activity, a technique of substrate zymography is commonly used. For MMP levels, ELISA, multiplex, or western blot analyses are applied. Unfortunately, terms “activities” and “concentrations” are not always correctly used in research articles. The term “MMP levels,” which should be related to MMP concentrations, is often used for both MMP activity and its concentration. It is also not rare to find comparisons between reported activities and concentrations without distinguishing their meaning in numerous published review articles. Thus, the reader has to search the method section to properly understand the MMP data provided in the corresponding paper. Notably, this may also account for the reported inconsistencies in MMP-focused AD research.

Regarding the MMP determination in various body fluids, CSF is the product of the nervous system; however, different tissues and systems are contributing to MMP activity/concentration in blood plasma (or serum). Plasma concentrations of MMPs are typically elevated in multiple diseases, e.g., cardiovascular [19, 106]. An increased age of the patient, which is a risk factor for AD development, is—at the same time—a risk factor for cardiovascular and other diseases. Therefore, an increase of MMP activity observed in an older patient with AD could be theoretically a consequence of heart or renal failure. It may at least partially explain the inconsistent findings, i.e., increased and decreased as well as unchanged MMP concentrations in AD patients reported by different research teams.

Focusing on the source of biological material for analyses, there is also an increased effort to apply less invasive methods to get information about some biomarkers, including the AD research and MMPs. MMPs could be determined, e.g., in saliva, as already suggested previously [107]. As mentioned previously, blood plasma may not be an optimal body fluid representing the situation in the nervous tissue; however, it is still possible to isolate extracellular vesicles derived from nervous tissue and determine MMP activities/concentrations therein. Despite being a more complicated procedure, it is still less invasive than a lumbar puncture to obtain a CSF sample.

## Conclusion

In addition to canonical pathways of APP processing, multiple studies showed that MMPs/TIMPs can also be involved in these processes as well as associated with the tau pathology, amyloid β metabolism, neuroinflammatory stimuli, and other factors contributing to the AD dementia. Although there is no exact consensus regarding the changes of MMP/TIMP activities and levels measured in blood or CSF of AD patients, MMPs are supposed to be indicative of tissue remodeling, progression of MCI to AD, and prognosis of patients suffering from AD. At the very minimum, their position in multimarker panel could be justified in order to predict the development and progression of AD.

## Data Availability

No datasets were generated or analysed during the current study.
